# Tracheobronchoplasty and Diaphragmatic Plication under VV ECMO for Combined ECAC and Diaphragmatic Paralysis

**DOI:** 10.1155/2021/5565754

**Published:** 2021-11-19

**Authors:** Mehmet M. Tatari, David Abia-Trujillo, Mathew Thomas, Neal M. Patel, Sebastian Fernandez-Bussy, Britney N. Hazelett, Margaret M. Johnson

**Affiliations:** ^1^Division of Pulmonary Medicine, Mayo Clinic, Jacksonville, FL. 4500 San Pablo Road, Jacksonville, FL 32224, USA; ^2^Department of Cardiothoracic Surgery, Mayo Clinic, Jacksonville, FL. 4500 San Pablo Road, Jacksonville, FL 32224, USA

## Abstract

The coexistence of expiratory central airway collapse and diaphragmatic paralysis presents a diagnostic and treatment challenge. Both entities are underrecognized causes of dyspnea, cough, sputum production, and orthopnea. Optimal treatment must be individualized and is best achieved by a multidisciplinary team. We present a case of a patient with profound functional impairment from dyspnea and hypoxemia due to expiratory central airway collapse, complicated by bronchiectasis from recurrent respiratory infections, and diaphragmatic paralysis.

## 1. Introduction

There are a myriad of causes of dyspnea, hypoxemia, and excessive sputum production. Identifying the relevant contributors to patient's presenting concerns is critical for appropriate management. Expiratory central airway collapse (ECAC) involving either the cartilaginous tracheal wall or the posterior membranous portion may be suggested by computed tomography imaging of the chest obtained during forced expiration and confirmed by dynamic bronchoscopy. Fluoroscopy and diaphragm ultrasound are used to diagnose diaphragmatic paralysis. Surgical management includes tracheobronchoplasty and diaphragmatic plication. ECAC and diaphragm paralysis both compromise gas exchange during surgery but the effect can be mitigated by the use of intraoperative extracorporeal membrane oxygenation (ECMO).

## 2. Case Presentation

A 54-year-old man presented with progressive shortness of breath, cough, and recurrent pneumonia. Chest examination was notable for reduced bilateral lung excursion, coarse breath sounds diffusely, and paradoxical breathing in the supine position. Pulmonary function testing suggested severe restriction with FVC of 27% predicted, FEV 1 28% predicted, normal FEV1/FVC ratio, and respiratory muscle strength < 10% predicted.

**Figure 1:**
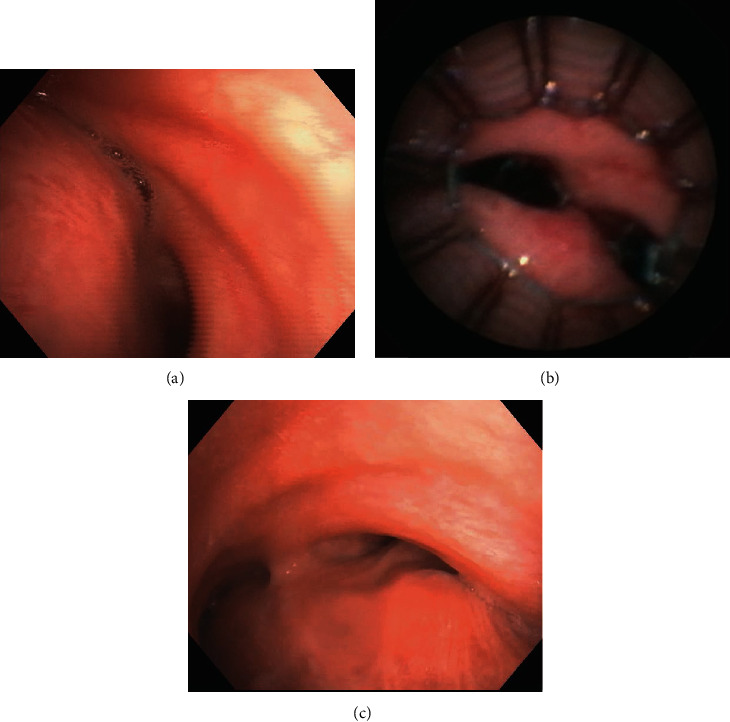
(a) Dynamic bronchoscopy showing severe expiratory central airway collapse of the distal trachea, left main stem bronchus, and right main bronchus. (b) Self-expandable metallic stents deployed in the airway during stent trial. (c) Dynamic bronchoscopy showing <70% collapse of the distal trachea, left main stem bronchus, and right main bronchus.

Computed tomography (CT) of the chest revealed basilar bronchiectasis and bilateral diaphragmatic elevation with associated atelectasis. Dynamic CT showed anterior displacement of the posterior tracheal wall at the distal trachea, bilateral main stem bronchi, and bronchus intermedius. Electromyogram demonstrated bilateral phrenic neuropathy and left brachial plexus neuropathy. Ultrasound confirmed the absence of diaphragm movement bilaterally. He had progressive clinical decline requiring near constant noninvasive positive pressure ventilation (NPPV). Dynamic bronchoscopy showed severe expiratory central airway collapse (ECAC) with >90% collapse of the trachea, main stem bronchi, and bronchus intermedius (Figure 1(a)). A stent trial (Figure 1(b)) was performed with placement of three uncovered self-expandable metallic stents in the left mainstem bronchus, distal and mid trachea and right mainstem bronchus and bronchus intermedius. This resulted in measurable improvements in the quality of life by validated questionnaires at two weeks and the stents were removed. He was recommended to undergo combined tracheobronchoplasty and right diaphragmatic plication.

The initial surgery was aborted due to the inability to maintain adequate oxygenation during single lung ventilation. One week later, the procedure was successfully completed through right thoracotomy under venous-venous extra-corporeal membrane oxygenation (VV ECMO) support. Central airway stabilization was achieved by suturing a knitted polypropylene mesh to the posterior membrane of the trachea and bilateral main bronchi. Due to persistent ventilator dependency, left diaphragm plication through a left thoracotomy was performed 5 days postoperatively. He was discharged home on postoperative day #23.

**Figure 2:**
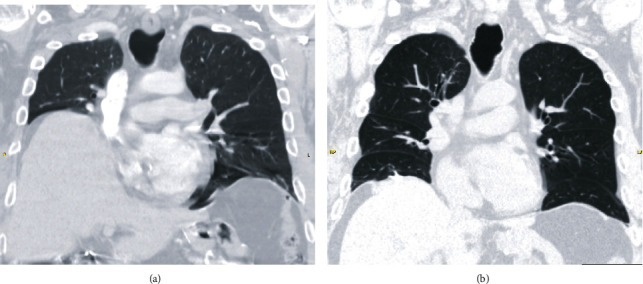
Coronal reconstruction of chest computed tomography (a) pre- and (b) postdiaphragmatic plication with noted improved lung expansion.

At follow-up evaluation, he no longer required NPPV and regained independence in activities of daily living. Pulmonary function testing showed improvement with FVC of 53% predicted, FEV1 42% predicted, and respiratory muscle strength 13% predicted. CT of the chest demonstrated improved lung volumes (Figure 2). Dynamic bronchoscopy showed improvement in the degree of ECAC (Figure 1(c)).

## 3. Discussion

**Figure 3:**
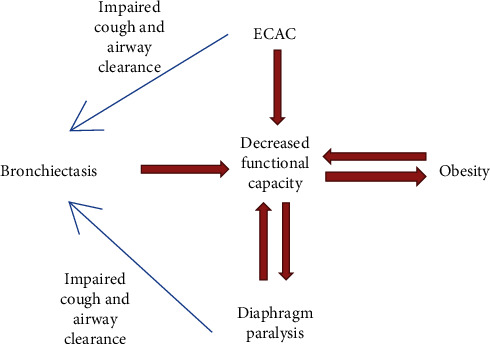
Interplay between conditions.

Although diagnostic parsimony encourages identifying a single unifying explanation for one's symptoms, this case illustrates the veracity of Hickam's dictum: “A man may have as many diseases as he damn well pleases [1].” We hypothesized that both ECAC and diaphragmatic paralysis (DP) were major contributors to his clinical presentation, and thus, therapy directed at each was mandated. Bronchiectasis was exacerbated by both conditions and the airway clearance therapy needed for bronchiectasis was precluded by the physiologic limitations imposed by coexistent ECAC and DP (Figure 3).

### 3.1. Diaphragm Dysfunction

Unilateral or bilateral dysfunction of the diaphragm can result from phrenic nerve dysfunction, neuromuscular junction disease, or myopathy. Paradoxical respiratory efforts and decline in FVC while supine suggest DP with confirmation achieved with fluoroscopy, ultrasound, or phrenic nerve EMG. Symptomatic unilateral disease that cannot be explained by another underlying disease process may be amenable to plication. Bilateral DP can be managed by noninvasive positive-pressure ventilation and treatment, if available, of the underlying cause. Diaphragmatic pacing may be beneficial if phrenic nerve function is intact. The goal of plication is at eliminating the paradoxical upward motion of the paralyzed diaphragm and decreasing the basilar parenchymal compression. Although historically performed via a thoracotomy, plication can also be performed effectively with minimally invasive robotic, thoracoscopic, or laparoscopic techniques [2]. Surgical plication of the diaphragm has shown a sustained improvement in FVC of 20% at 48 months [2, 3].

### 3.2. Expiratory Central Airway Collapse (ECAC)

Recognition of large airway dysfunction during expiration as a cause of chronic respiratory symptoms is gradually increasing. Two patterns have been described in ECAC: (1) tracheobronchomalacia (TBM) in which the cartilaginous component of the anterolateral tracheal wall collapses on forced expiration and (2) excessive dynamic airway collapse (EDAC) in which there is anterior displacement of the posterior (membranous) tracheal wall during expiration [4]. When clinically suspected, dynamic images of the chest obtained during forced expiration can suggest ECAC. Dynamic bronchoscopy during which the degree of expiratory compromise is estimated during spontaneous awake breathing remains the diagnostic gold standard. Nonsevere disease can be treated conservatively with measures to improve bronchial hygiene, weight loss, treatment of coexisting diseases, and intermittent use of positive airway pressure.

When the degree of airway collapse is severe, surgical repair should be considered. A preoperative stent trial with uncovered self-expandable metallic stents is strongly advised to estimate the potential surgical benefit prior to surgical intervention [4]. In our patient, we used three uncovered stents to preserve mucociliary function and prevent the mucus plugging seen with silicone stents. Also, self-expandable metallic stents adapt better than silicone stents to any airway irregularity. Since these uncovered stents are removed 7–10 days later, they are not embedded into the airway mucosa [5].

Our technique for tracheobronchoplasty involves plication of the redundant posterior membrane of the trachea and mainstem bronchi and fixation to a semirigid permanent (polypropylene) permeable mesh. The standard approach is through a right thoracotomy, although minimally invasive robotic and thoracoscopic approaches have also been reported to be feasible but with unknown long-term results [6].

### 3.3. Bronchiectasis

We hypothesize that basilar bronchiectasis in this patient was due to inadequate airway clearance from both ECAC and DP resulting in recurrent lower respiratory infections. Medical management of bronchiectasis to improve airway mucous clearance was limited due to compromised large airways from ECAC and weak cough from DP.

### 3.4. Extracorporeal Membrane Oxygenation

VV ECMO is used most commonly in acute respiratory distress syndrome and in patients with advanced lung disease requiring transplant either intraoperatively or as a bridge to lung transplantation. As the use of ECMO increases, understanding the surgical implications of this therapy is critical. In a review of 563 patients on ECMO, 269 required noncardiac surgery. There was no difference in outcomes between those needing or not needing surgery [7]. Anticipatory use of ECMO to facilitate intraoperative support for a patient with pulmonary compromise holds promise. Preemptive use of VV ECMO has been reported to support surgical repair of critical central airway obstruction and tumors [8, 9].

Both ECAC and bilateral diaphragmatic dysfunction, with associated bronchiectasis, synergistically lead to dyspnea, cough, respiratory infections, and severe functional impairment in this patient. Demonstration of the improvement in clinical outcomes with an airway stent trial before entertaining surgical correction of ECAC is critical to optimize patient selection. As demonstrated, surgical intervention for both ECAC and diaphragm paralysis can dramatically improve the quality of life and functional capability. Intraoperative ECMO may permit definitive surgical treatment of ECAC in patients with gas exchange abnormalities that would otherwise preclude surgery.

This case elucidates three critical concepts to comprehensive patient-centered care. The importance of thoroughly investigating all the relevant contributors to a patient's symptoms, with specific attention to those for which therapy can be offered, is illustrated. Additionally, the novel use of ECMO demonstrates the need to embrace unconventional surgical approaches as mandated by the patient's clinical scenario. Finally, it was only the multidisciplinary team-based approach to care, involving pulmonologists, interventionalists, thoracic surgeons, anesthesiologists, and intensivists that allowed successful management of this patient's complex presentation.
